# HIV drug resistance in patients in China’s national HIV treatment programme who have been on first-line ART for at least 9 months

**DOI:** 10.1186/s12981-020-00264-5

**Published:** 2020-03-05

**Authors:** Pengtao Liu, Hui Xing, Lingjie Liao, Yi Feng, Xuebing Leng, Jing Wang, Wei Kan, Jing Yan, Yang Li, Zhongbao Zuo, Yinghui You, Yuhua Ruan, Yiming Shao

**Affiliations:** 1 Weifang Medical University, Weifang, 261053 Shandong China; 2 State Key Laboratory for Infectious Disease Prevention and Control (SKLID), National Center for AIDS/STD Control and Prevention (NCAIDS), Chinese Center for Disease Control and Prevention (China CDC), Collaborative Innovation Center for Diagnosis and Treatment of Infectious Diseases, 155 Changbai Road, Changping District, Beijing, 102206 People’s Republic of China; 3 Guangxi Zhuang Autonomous Region Center for Disease Control and Prevention, Nanning, Guangxi China

**Keywords:** HIV-1, Drug resistance, ART, NNRTIs, NRTIs

## Abstract

**Background:**

The aim of this study was to assess trends in drug resistance and associated clinical and programmatic factors at a national level during the rapid scale up of ART.

**Methods:**

Logistic regression was used to identify the factors associated with HIVDR. Variables associated with drug resistance in multivariable logistic regression were included in the Cochran–Armitage test for trend.

**Results:**

A total of 11,976 patients were enrolled in the study. The prevalence of HIVDR among patients who received ART for 9–24 months during 2003–2008, 2009–2012, and 2013–2015 significantly decreased (15.5%, 6.3%, and 2.3%, respectively, *P* < 0.01). With respect to the class of antiretroviral, there were substantial increases in resistance to both non-nucleoside reverse transcriptase inhibitors (NNRTIs) and nucleoside reverse transcriptase inhibitors (NRTIs) (2003–2008, 2009–2012, and 2013–2015: 49.7%, 58.9%, and 73.0%, respectively, *P* < 0.01). The prevalence of DR to protease inhibitors (PIs) was low, which supported their continued use as second-line therapy in China.

**Conclusions:**

Our results provide evidence for the effectiveness of China’s “Treat All” approach to guide policy makers to improve training for healthcare providers and education on ART adherence among patients.

## Background

The global expansion in the use of antiretroviral therapy (ART) has markedly reduced HIV-related morbidities, mortalities, disease transmission, and adult HIV incidence [1,2]. However, the emergence and spread of high levels of HIV-1 drug resistance (HIVDR) in low- and middle-income countries (LMICs), where a marked increase in the use of combined antiretroviral treatment has occurred, could compromise the effectiveness of national HIV treatment programs [2–4]. Recent studies showed that the HIVDR rates were 21% in Namibia, 18.8% in the African regions, and 15.0% in the Southern African regions [5].

With the continued roll-out of ART in LMICs, evidence is emerging of increasing levels of drug resistance prevalence [5, 6]. To extend the benefits of ART to resource-limited settings, which bear most of the HIV burden [7], the World Health Organization (WHO) encourages country ownership through national HIVDR working groups, plans and strategic investment. In some LMICs, national health systems have limited infrastructure, a shortage of healthcare professionals, and weak enforcement of quality standards [8]. These conditions parallel the ones in China 10 years previously. However, throughout the period of increasing implementation of ART, China’s National Free Antiretroviral Treatment Program (NFATP) has been largely successful, with an overall reduction in virological failure and HIV drug resistance since 2008 [9]. Although there is limited access to many antiretrovirals (ARVs) in China, data from the NFATP study prove that understanding the scientific basis and clinical implications of HIVDR is useful in order to shape appropriate surveillance, inform treatment algorithms, and manage difficult cases [9–12].

Three phases of ART were established according to development stages of NFATP in China: initial and scale-up ART, 2003–2008; standardization and scale-up, 2009–2012; and rapid scale-up, 2013–2015 [13–15]. Enormous efforts have been made to develop the capability of treating large numbers of people across wide geographic areas. Important policy changes included increased HIV testing among at-risk populations, as well as an update to the National Free ART Guideline to include patients with higher CD4 cell counts or WHO stage I/II disease and to replace didanosine (ddI) with lamivudine (3TC) in the standard first-line ART regimen in order to significantly decrease HIV-related mortality and HIV transmission. The aim of this study was to evaluate the trends of HIV drug resistance prevalence in treated individuals since the initiation of ART in China. We also examined the effects of the NFATP and the changes in potential clinical and programmatic factors over the past 10 years.

## Methods

### Study design and study participants

Data collected through the Chinese National HIVDR Surveillance and Monitoring Network since 2003 were analyzed using a retrospective method to assess HIVDR and associated program factors in populations of patients on ART. The database included annual cross-sectional surveys of HIV drug resistance from 2003 to 2015 in China which were described in the previous studies [9, 10, 14, 16–18]. and the methodology was endorsed by WHO for HIVDR Monitoring Survey [6,19]. The eligibility criteria were as follows: [1] age ≥ 18 years; [2] receipt of national free initial first-line ART for ≥ 9 months of treatment; [3] visit to a participating clinic for routine NFATP; [4] blood specimens collected for viral load (VL) testing; and [5] consent and willingness to participate in the study. By the end of December 2015, 11,976 cases (ART for at least 9 months) met the inclusion criteria, which represented 2.5% of the 471,140 patients treated by the NFATP. 4679 participants (ART for 9–24 months) were selected in order to compare with other studies and facilitate follow-up treatment.

### Laboratory tests

Blood samples of the participants were collected by the local CDC at enrolment and every follow-up visit. Within 8 h, blood samples were sent at ambient temperature to the laboratory of NCAIDS in Beijing, where a team was on-call 24 h for 7 days a week for receipt. Within 2 h after arriving at the laboratory, CD4 cell count was conducted using flow cytometry (FACS Calibur, BD Company, Franklin Lakes, New Jersey, USA). Plasma was isolated and frozen at − 80 °C until testing for viral load and HIVDR. According to the manufacturers’ recommendations, plasma HIV RNA in this study was quantified with real-time NASBA (NucliSense Easy Q, bioMe′rieux, Lyon, France) or with Amplicor HIV-1 monitor test (COBAS, Roche Applied Science, Penzberg, Germany). A VL ≥ 1000 was classified as virologic failure based on WHO HIVDR surveillance guideline. In samples with virologic failure on ART, HIVDR genotyping was performed at the WHO Accreditation Laboratory of NCAIDS, China CDC by using an in-house method as previously described [9, 17, 20, 21]. Drug resistance mutation analysis and viral subtype determination were performed on a 1.3 kb section of the HIV pol gene using the Stanford University HIVDR Database online sequence analysis tool (http://hivdb.stanford.edu/, accessed 2 November 2018). All HIVDR mutations that conferred low (total score 15 to 29), intermediate (total score 30 to 59) or high-level (total score ≥ 60) resistance were included [9, 22]. The frequency of resistance to specific ARV drugs and resistance to both NRTI and NNRTI drugs was calculated by SAS program according to the results of the Stanford online sequence analysis tool. Study participants were informed of the laboratory tests results and the results were used to select subsequent ART regimen.

### Statistical analysis

Logistic regression was used to identify the factors associated with HIVDR. The factors associated with HIVDR that were significant in the univariate logistic regression analysis were included in the multivariate logistic regression. Variables associated with resistance in the multivariable logistic regression model and those clinically meaningful were included in the Cochran–Armitage test for trend. Somers’ D (R|C) was used to measure the association between the variables (considered as the response) and the time of initial ART (year; considered as a predictor). Statistical significance was defined as a *P* value < 0.05. All the statistical analyses were performed with SAS 9.4 software (SAS Institute, Cary, NC, USA).

## Results

### Factors associated with drug resistance among patients who received first-line ART

Among the 4679 patients who received first-line ART for 9–24 months, 8.6% (402) patients had HIVDR. The prevalence of HIVDR during 2003–2008 (n = 1882), 2009–2012 (n = 1163), and 2013–2015 (n = 1634) significantly decreased (15.5%, 6.3%, and 2.3%, respectively, Cochran-Armitage test, *P* < 0.01). The factors significantly associated with HIVDR in the multivariate logistic regression model were education, occupation, route of HIV infection, WHO clinical stage III/IV, CD4 cell count before ART, and time of beginning ART. Compared with patients with post-secondary school or higher education, those with secondary school or lower education were more likely to show DR (adjusted odds ratio [AOR]: 1.5, 95% confidence interval [CI] 1.0–2.1). Compared with people from other occupations, farmers were more likely to show DR (AOR: 1.8, 95% CI 1.3–2.5). With regard to route of HIV infection, compared with heterosexual intercourse, homosexual intercourse was likely to show less DR (AOR: 0.8, 95% CI 0.4–1.5); blood or plasma transfusion was more likely to show DR (AOR: 2.9, 95% CI 2.0–4.4); intravenous drug use was more likely to show DR (AOR: 2.1, 95% CI 1.4–3.1); and other or unknown routes were more likely to show DR (AOR: 1.8, 95% CI 1.1–3.1). Furthermore, compared with patients with other WHO clinical stages, those with WHO clinical stage III/IV were more likely to show DR (AOR: 1.6, 95% CI 1.1–2.1). Patients with CD4 count ≥ 349 cells/mm^3^ showed less DR than patients with CD4 count < 349 cells/mm^3^ (AOR: 0.6, 95% CI 0.3–0.9), and patients who began ART from or after 2009 showed less DR than patients who began ART before 2009 (AOR: 0.5, 95% CI 0.4–0.7). With regard to the location of ART drug distribution, county hospital or CDC showed lower DR than township hospital or village clinic (AOR: 0.4, 95% CI 0.3–0.5). Patients who missed any dose in the past month were more likely to show DR than those who did not miss any dose (AOR: 1.7, 95% CI 1.1–2.5). Compared with lamivudine-based regimens, other regimens were more likely to show DR (AOR: 1.6, 95% CI 1.2–2.1) (Table 1).

**Table 1:** Factors associated with drug resistance among Chinese patients who commenced first-line ART for 9–24 months from 2003 to 2015 (n = 4679)

Factors	Total	DR	%	OR (95% CI)	P	AOR* (95% CI)	P
Total	4679	402	8.6				
Sex							
Male	3177	254	8.0	1.0			
Female	1502	148	9.9	1.3 (1.0,1.6)	0.03		
Age (years)							
≤ 30	921	56	6.1	1.0			
31–40	1731	178	10.3	1.8 (1.3,2.4)	< 0.01		
> 40	2027	168	8.3	1.4 (1.0,1.9)	0.67		
Education							
Post-secondary school or more	1424	59	4.1	1.0			
Secondary school or less	3255	343	10.5	2.7 (2.1,3.6)	< 0.01	1.5 (1.0,2.1)	0.04
Occupation							
Other	2218	96	4.3	1.0			
Farmer	2409	301	12.5	3.2 (2.5,4.0)	< 0.01	1.8 (1.3,2.5)	<0.01
Marital status							
Other	3228	332	10.3	1.0			
Married	1451	70	4.8	0.4 (0.3,0.6)	< 0.01		
Time of initial ART (year)							
≤ 2008	1882	292	15.5	1.0		1.0	
> 2008	2797	110	3.9	0.2 (0.2,0.3)	< 0.01	0.5 (0.4,0.7)	< 0.01
Route of HIV infection							
Heterosexual intercourse	2166	95	4.4	1.0		1.0	
Homosexual intercourse	670	15	2.2	0.5 (0.3,0.9)	< 0.01	0.9 (0.5,1.7)	0.06
Blood/IDU	1531	266	17.4	4.6 (3.6,5.9)	< 0.01	2.4 (1.8,3.3)	< 0.01
Other or unknown	312	26	8.3	2.0 (1.3,3.1)	0.07	1.8 (1.1,3.1)	0.22
WHO clinic stage III/IV before ART							
No	3842	283	7.4	1.0		1.0	
Yes	837	119	14.2	2.1 (1.7,2.6)	< 0.01	1.6 (1.1,2.1)	< 0.01
CD4 cell counts before ART (cells/mm^3^)							
< 349	3579	263	7.3	1.0		1.0	
≥ 350	631	25	4.0	0.5 (0.3,0.8)	< 0.01	0.6 (0.3,0.9)	0.01
Initial ART regimens							
D4T/3TC/EFV or NVP	1053	87	8.3	1.0		1.0	
AZT/3TC/EFV or NVP	1901	95	5.0	0.6 (0.4, 0.8)	< 0.01	0.7 (0.5, 1.0)	<0.01
TDF/3TC/EFV or NVP	985	36	3.7	0.4 (0.3, 0.6)	< 0.01	0.5 (0.3, 0.8)	< 0.01
AZT/ddI/EFV or NVP	482	153	31.7	5.2 (3.9, 6.9)	< 0.01	6.3 (4.2, 9.4)	< 0.01
Other regimens	258	31	12.0	1.5 (1.0, 2.3)	0.08	1.0 (0.5, 2.0)	0.55
ART regimens at survey							
D4T/3TC/EFV or NVP	605	32	5.3	1.0			
AZT/3TC/EFV or NVP	1774	73	4.1	0.8 (0.5, 1.2)	< 0.01	0.9 (0.6, 1.4)	< 0.01
TDF/3TC/EFV or NVP	1165	39	3.4	0.6 (0.4, 1.0)	< 0.01	0.8 (0.5, 1.3)	< 0.01
Other first-line regimens	683	147	21.5	4.9 (3.3, 7.3)	< 0.01	4.7 (2.9, 7.7)	< 0.01
Second-line regimens	452	111	24.6	5.8 (3.8, 8.8)	< 0.01	5.6 (3.5, 8.9)	< 0.01
Missed doses in the past month							
No	4316	353	8.2	1.0			
Yes	363	49	13.5	1.8 (1.3,2.4)	< 0.01	1.7 (1.1,2.5)	0.01
ART drug distribution location							
Township hospital or village clinic	2564	324	12.6	1.0		1.0	
County hospital or CDC	2110	78	3.7	0.3 (0.2,0.3)	< 0.01	0.4 (0.3,0.5)	< 0.01
Adverse drug reactions							
No	3842	283	7.4	1.0		1.0	
Yes	837	119	14.2	2.1 (1.7,2.6)	< 0.01	1.6 (1.2,2.1)	< 0.01

^*^ Adjusted for: sex, age, education, marital status, occupation, and year of initial ART

### Changes in factors associated with drug resistance in patients who commenced first-line ART during 2003–2008, 2009–2012, and 2013–2015

Variables associated with HIVDR in the multivariable regression model were included in the Cochran–Armitage test for trend over time. These variables included education, occupation, marital status, route of HIV infection, CD4 cell count before ART, initial ART regimen, missed doses in the past month, location of ART drug distribution, and adverse drug reactions. The proportions of these variables were compared among patients who received ART during 2003–2008, 2009–2012, and 2013–2015. A trend test showed statistically significant changes in factors associated with drug resistance over time for: education level of high school and above, farming occupation, heterosexual or homosexual intercourse, WHO clinical stage III/IV before ART, CD4 cell count ≥ 350 before ART, lamivudine-based regimens, drug receipt from a county hospital or CDC, and adverse drug reactions. However, there was no significant change related to missed doses in the past months (Table 2).

**Table 2:** Changes in factors associated with drug resistance in patients who commenced first-line ART during 2003–2008, 2009–2012, and 2013–2015 (n = 11,976)

Factors	2003–2008 (%)		2009–2012 (%)		2013–2015 (%)		
	N	%	N	%	N	%	P*
Total	7595	100	2567	100	1814	100	
Education							< 0.01
Secondary school or less	6494	85.5	1835	71.5	1022	56.3	
High school and above	1101	14.5	732	28.5	792	43.7	
Occupation							< 0.01
Other	2274	30.1	1231	48.3	964	53.8	
Farmer	5277	69.9	1320	51.7	829	46.2	
Route of HIV infection [1]							< 0.01
Heterosexual/homosexual intercourse	2399	31.6	1212	47.2	961	53.0	
Other	5196	68.4	1355	52.8	853	47.0	
Route of HIV infection [2]							< 0.01
Blood/IDU	4780	62.9	906	35.3	121	6.67	
Other	2815	37.1	1661	64.7	1693	93.3	
WHO clinic stage before ART							< 0.01
No	5253	69.2	1945	75.8	1715	94.5	
Yes	2342	30.8	622	24.2	99	5.5	
CD4 cell counts before ART (cells/mm^3^)							< 0.01
< 349	5664	90.8	2144	89.1	1388	78.0	
≥ 350	572	9.2	263	10.9	392	22.0	
Initial ART regimens [1]							< 0.01
3TC- based regimens	4991	65.7	1934	75.3	1424	78.5	
Other regimens	2604	34.3	633	24.7	390	21.5	
Initial ART regimens [2]							< 0.01
D4T/3TC/EFV or NVP	2863	37.7	607	23.7	81	4.5	
Other regimens	4732	62.3	1960	76.3	1733	95.5	
Initial ART regimens [3]							< 0.01
TDF/3TC/EFV	83	1.1	239	9.3	868	47.9	
Other regimens	7512	98.9	2328	90.7	946	52.1	
Missed doses in the past month							0.18
No	6925	91.2	2363	92.0	1674	92.3	
Yes	670	8.8	204	8.0	140	7.7	
ART drug distribution location							< 0.01
Township hospital or village clinic	6775	89.5	1463	57.2	97	5.4	
County hospital or CDC	791	10.5	544	42.8	1717	94.6	
Adverse drug reactions							< 0.01
No	5095	67.1	1945	75.8	1715	94.5	
Yes	2500	32.9	622	24.2	99	5.5	

* Cochran-Armitage Trend Test, two-sided

### HIVDR mutations

There were substantial increases in resistance to both NNRTI and NRTIs (2003–2008, 2009–2012, and 2013–2015: 49.7%, 58.9%, and 73.0%, respectively, *P* < 0.01). The most-common NNRTI mutations occurred at positions 101 (9.5%), 103 (49.5%), 181 (12.9%), and 190 (9.5%) in the RT region; the most-common NRTI mutations occurred at codons 41 (10.2%), 67 (13.4%), and 184 (32.3%) in the RT region; and protease inhibitors (PI) mutations were most frequently seen at codons 32 (0.7%), 46(1.5%) and 54 (0.7%) in the protease (PR) region (Table 3).

**Table 3:** HIV drug resistance mutations among Chinese patients with acquired drug resistance from 2003 to 2015 (9–24 months of first-line ART)

Antiretroviral drugs	Total		2003–2008		2009–2012		2013–2015		Resistance mutations (%)
	N	%	N	%	N	%	N	%	
Total	402	100.0	292	100.0	73	100.0	37	100.0	V90I/V (3.5), A98G (2.2), L100I (1.0), K101E/N (9.5), K103N (49.5), V106A/M/I (9.2), V108I (2.7), E138A/G/K (1.7), V179D/T/E (6.0), V181C/I/V (12.9), Y188F/H/L/Y (5.2), G190A/S/R (9.5), H221Y (0.7), P225H (1.7), F227L (2.5), M230L (1.5), K238N/T (1.0)
NNRTI and NRTI	215	53.5	145	49.7	43	58.9	27	73.0	
NNRTI (any)	338	84.1	235	80.5	68	93.2	35	94.6	
Efavirenz (EFV)*	334	83.1	232	79.5	67	91.8	35	94.6	
Nevirapine (NVP)*	337	83.8	235	80.5	67	91.8	35	94.6	
Delavirdine (DLV)	231	57.5	215	73.6	16	21.9	0	0.0	
Etravirine (ETV)	70	17.4	30	10.3	19	26.0	21	56.8	
Rilpivirine (RPV)	47	11.7	0	0.0	23	31.5	24	64.9	
NRTI (any)	271	67.4	200	68.5	44	60.3	27	73.0	M41L (10.2), A62V (1.0), K65R (8.5), D67N/G/DN (13.4), T69N (5.0), K70E/R (4.0), L74I/V (3.0), V75A/M/T (2.2), Y115F (2.5), M184I/V (32.3), T215C/D/F/I/S/Y (7.2), K219E (2.2)
Emtricitabine (FTC)	198	49.3	130	44.5	43	58.9	25	67.6	
Lamivudine (3TC)*	197	49.0	129	44.2	43	58.9	25	67.6	
Abacavir (ABC)	195	48.5	126	43.2	43	58.9	26	70.3	
Didanosine (ddI)*	171	42.5	126	43.2	23	31.5	22	59.5	
Stavudine (d4T)*	162	40.3	125	42.8	18	24.7	19	51.4	
Tenofovir (TDF)*	129	32.1	96	32.9	15	20.5	18	48.6	
Azidothymidine (AZT)*	126	31.3	114	39.0	8	11.0	4	10.8	
PI (any)	20	5.0	6	2.1	10	13.7	4	10.8	V32L/E (0.7), M46I (1.5), G48E (0.2), I54V (0.7), L76S (0.2), G73C/S (0.1), L90M/S (0.5)
Tipranavir (TPV)	7	1.7	4	1.4	3	4.1	0	0.0	
Fosamprenavir (FPV)	5	1.2	2	0.7	3	4.1	0	0.0	
Lopinavir (LPV)*	3	0.7	1	0.3	2	2.7	0	0.0	
Nelfinavir (NFV)	15	3.7	4	1.4	7	9.6	4	10.8	
Atazanavir (ATV)	6	1.5	3	1.0	3	4.1	0	0.0	
Darunavir (DRV)	2	0.5	1	0.3	1	1.4	0	0.0	
Indinavir (IDV)	6	1.5	3	1.0	3	4.1	0	0.0	
Saquinavir (SQV)	6	1.5	3	1.0	3	4.1	0	0.0	

*Provided through the National Free Antiretroviral Treatment Program (NFATP)

## Discussion

This retrospective analysis of the Chinese National HIVDR Surveillance Database from 2003 to 2015 showed the overall prevalence of HIVDR has declined significantly since the commencement of programs for ART use in China (2018). The recent (2009–2012, 2013–2015) prevalence of HIVDR among patients who received first-line ART for 9–24 months was found to be 6.3 and 2.3%, respectively. The prevalence of HIVDR found in our study was significantly lower than that in some developed countries or countries where free ART was available nationwide [3, 23]. The drug resistance prevalence was found to be relatively low in China, as compared with the prevalence in other LMICs, even after the rapid scale up of ART [3, 24].

The overall low prevalence of DR may reflect the relatively low coverage and short duration of free ART or, equally, may reflect the success of first-line ART regimens and HIV prevention programs. Our study assessed the clinical and programmatic factors associated with HIVDR in patients who received first-line ART during 2003–2015. Virologic failure among Chinese patients who received ART for 9–24 months during 2003–2008, 2009–2012, and 2013–2015 significantly improved (25.7%, 17.3%, and 7.0%, respectively, P < 0.01).The significant improvements in virologic outcome in Chinese patients receiving ART treatment over the past 10 years may be attributable to changes in a number of clinical and programmatic factors. Firstly, the Chinese NFATP policy has changed since 2003, due to the availability of an expanded range of antiviral drug resources. For instance, AZT, d4T, ddI, and NVP were generically produced in China and used as standard prescriptions from the beginning of the NFATP, whereas 3TC and EFV were branded drugs that became available in 2005 and were gradually more commonly prescribed. First-line ART regimens were revised to consist of tenofovir (TDF)/AZT + EFV/NVP in 2008 [9]. Our previous study showed that the decline in virologic failure and HIVDR were strongly associated with the standardized use of 3TC-based regimens instead of ddI-based regimens; this study confirmed the previous finding, showing since 2008, virological failure at 12–15 months of treatment had improved from 26.6 to 12.1%, and HIVDR rates were also significantly decreased from 15.4 to 5.4% [9]. Our studies also showed that patients who initiated 3TC-based regimens showed improvements in adherence compared with those who initiated ddI-based regimens. 3TC-based regimens have more curative effect than ddI-based regimens, and it is advantageous in improving the patient’s quality of life and the compliance of medicine. Replacing ddI-based regimens may explain some of the difference seen in terms of the tolerability in the treatment population, and this improvement could greatly improve adherence and subsequently less HIVDR. Secondly, adverse effects were reduced from 2003 to 2015 due to a revision of the first-line ART regimen in China, since 2003 patients had started ART when the CD4 cell count was < 200 cells/mm^3^; this threshold then increased to 350 cells/mm^3^ in 2012 and further increased to 500 cells/mm^3^ in 2014. A further important policy change in NFATP included the location of ART drug distribution; increased rates of distribution at county hospitals or CDC have occurred over time, which has improved the quality of care and treatment. The stratified analysis showed that patients receiving ART at county hospitals had lower HIVDR rates than those treated at village- or town-level clinics.

Changes in social and demographic factors among patients since the initiation of NFATP in 2003 had also been associated with treatment outcome over time. The proportion of HIV patients with a higher education level (high school and above) increased from 14.5% in 2003–2008 to 43.7% in 2013–2015. In addition, more HIV patients were from non-agricultural occupations (increased from 30.1 to 53.8%) and the number that had contracted HIV due to heterosexual or homosexual intercourse, compared to other routes such as drug use, increased from 31.6 to 53.0% (Table 2). Our previous studies showed that a low education level, the occupation of farming, drug use, and being a blood recipient were associated with a high prevalence of HIVDR [9, 18.] Although a significant change in these factors may have substantially contributed to the overall reduction in HIVDR in China, patient adherence (non-missed doses in the past month) did not significantly improve over time. Due to the rapidly increasing number of patients treated through the NFATP, more drug resistance could emerge in the future, especially if treatment adherence is not sustained. Therefore, it is crucial for the NFATP to focus on improving the quality of care and ART education in its next phases of implementation.

Rapid expansion of ART raises concerns for long-term sustainability and emerging dual-class resistance (NNRT and NRTIs) in China. Among the 402 treatment-experienced patients with DR mutations, 215 (53.5%) harbored dual-class resistance. With respect to the class of ART drugs, there were substantial increases in resistance to both NNRT and NRTIs over time (from 49.7 to 73.0%). In particular, expanded ART without improved adherence is likely to result in dual-class resistance, which may lead to poor treatment outcomes and HIVDR. The most common NNRTI mutations were K103 and Y181, and the most common NRTI mutations were M184, D67 and M41. In addition, the prevalence of DR to PIs was low, which supported their continued use as second-line therapy in China.

Apart from important findings, our study has several limitations. First, unlike a cohort study, in which a group of patients is assessed continuously over time, a cross-sectional survey, by its very nature, excludes patients who are no longer receiving ART and therefore cannot be observed because they have died, been lost to follow-up, or have stopped treatment. This survivor bias can significantly impact the interpretation of outcomes. In order to adjust for such a possible bias, we collected nationally representative data on retention at 12 months. By the end of 2015, 10.2% of the patients had died, whilst 8.7% were found to have terminated treatment among those treated by the NFATP in China. Accordingly, the adjusted prevalence of virologic failure and DR among patients who received first-line ART for 9–24 months during 2013–2015 was 7.0–8.3 and 2.3–2.7%, respectively. Second, a large proportion of patients began initial ddI treatment during the earlier years of the NFATP, when the baseline CD4 cell count was not required to be of a certain threshold and the standards of care and follow-up were low, which might have contributed to the poor treatment outcomes in the first few years of the NFATP.

## Conclusions

However, these limitations do not have a considerable bearing on our conclusion on the changes in HIVDR from 2003 to 2015 in China. To our knowledge, this is the first large observational study worldwide to assess the outcomes of ART over the past 10 years. The results provide evidence for the effectiveness of China’s HIV control and prevention efforts such as the “Treat All” approach. Furthermore, the findings will help guide policy makers to improve training for healthcare providers and enhance patient education on ART adherence. Further monitoring of virologic outcomes is needed to elucidate the determinants of long-term programmatic success. Government advocacy, specialist physician management, laboratory monitoring and constantly raising the level of education, all played a role in the success of NFATP. This study had the advantage of having thoroughly examined these factors and their relationship in one analysis, and the results were particularly important for countries with limited resources.

## Data Availability

The datasets used or analyzed during the current study are available from the corresponding author on reasonable request.

## Abbreviations

ART: Antiretroviral therapy
HIV: Human immunodeficiency virus
HIVDR: HIV drug resistance
NNRTIs: Non-nucleoside reverse transcriptase inhibitors
NRTIs: Nucleoside reverse transcriptase inhibitors
PIs: Protease inhibitors
LMICs: Low- and middle-income countries
WHO: World Health Organization
NFATP: National Free Antiretroviral Treatment Program
ARVs: Antiretrovirals
ddI: Didanosine
3TC: Lamivudine
D4T: Stavudine
AZT: Zidovudine
TDF: Tenofovir
CDC: Centers for disease control
